# Polymorphisms in the mitochondrial oxidative phosphorylation chain genes as prognostic markers for colorectal cancer

**DOI:** 10.1186/1471-2350-13-31

**Published:** 2012-04-30

**Authors:** Jesus Lascorz, Melanie Bevier, Witigo V Schönfels, Holger Kalthoff, Heiko Aselmann, Jan Beckmann, Jan Egberts, Stephan Buch, Thomas Becker, Stefan Schreiber, Jochen Hampe, Kari Hemminki, Asta Försti, Clemens Schafmayer

**Affiliations:** 1 Division of Molecular Genetic Epidemiology, German Cancer Research Center (DKFZ), Im Neuenheimer Feld 580, Heidelberg, 69120, Germany; 2 Department of General and Thoracic Surgery, Christian-Albrechts-University, Kiel, Germany; 3 Division of Molecular Oncology, Institute for Experimental Cancer Research, Christian-Albrechts-University, Kiel, Germany; 4 POPGEN Biobank Project, Christian-Albrechts-University, Kiel, Germany; 5 Department of General Internal Medicine, Christian-Albrechts-University, Kiel, Germany; 6 Center for Primary Health Care Research, Clinical Research Center, Lund University, Malmö, Sweden

## Abstract

**Background:**

Currently, the TNM classification of malignant tumours based on clinicopathological staging remains the standard for colorectal cancer (CRC) prognostication. Recently, we identified the mitochondrial oxidative phosphorylation chain as a consistently overrepresented category in the published gene expression profiling (GEP) studies on CRC prognosis.

**Methods:**

We evaluated associations of putative regulatory single nucleotide polymorphisms (SNPs) in genes from the oxidative phosphorylation chain with survival and disease prognosis in 613 CRC patients from Northern Germany (PopGen cohort).

**Results:**

Two SNPs in the 3′ untranslated region of UQCRB (complex III), rs7836698 and rs10504961, were associated with overall survival (HR = 0.52, 95% CI 0.32–0.85 and HR = 0.64, 95% CI 0.42–0.99, for TT carriers). These associations were restricted to the group of patients with cancer located in the colon (HR = 0.42, 95% CI 0.22–0.82 and HR = 0.46, 95% CI 0.25–0.83). Multivariate analysis indicated that both markers might act as independent prognostic markers. Additionally, the TT carriers were ~2 times more likely to develop tumours in the colon than in the rectum. Two SNPs in COX6B1 (complex IV) were associated with lymph node metastasis in a dominant model (rs6510502, OR = 1.75, 95% CI 1.20–2.57; rs10420252, OR = 1.68, 95% CI 1.11–2.53); rs6510502 was associated also with distant metastasis (OR = 1.67, 95% CI 1.09–2.56 in a dominant model).

**Conclusions:**

This is the first report suggesting that markers in genes from the mitochondrial oxidative chain might be prognostic factors for CRC. Additional studies replicating the presented findings are needed.

## Background

Colorectal cancer (CRC) is the third most common cancer and the fourth-leading cause of cancer death worldwide [1]. Currently, the TNM classification of malignant tumours based on clinicopathological staging remains the standard for CRC prognostication [2]. Many gene expression profiling (GEP) studies on prognosis of CRC have been performed in the last decade using microarray technology, with the aim to identify a gene expression profile to discriminate more aggressive from less aggressive CRC. However, due to their low overlap in the identified genes, no reliable signature useful in the clinical practice has been found. Recently, we published a systematic pathway-based enrichment analysis of 23 independent GEP studies on prognosis of CRC [3]. This analysis indicated the mitochondrial oxidative phosphorylation (OXPHOS) chain as a significantly and consistently overrepresented prognostic category for CRC.

Already several decades ago, it was suggested that impaired oxidative metabolism may cause malignant growth [4]. In cancer cells, there is an enhanced glucose use, slowing the rate of the tricarboxylic acid cycle and oxidative phosphorylation and increasing glycolysis, as the way to generate energy in form of adenosine triphosphate (ATP), despite aerobic conditions [5]. This assumption, known as Warburg’s hypothesis, has been rediscovered by a broad range of experimental approaches showing interaction of mitochondrial metabolism and tumour growth [6-8]. Additionally, germline mutations in the *mitochondrial succinate dehydrogenase* (complex II of the OXPHOS chain) subunits SDHD, SDHC, and SDHB are a frequent cause of paragangliomas of the head and neck and of phaeochromocytomas [9].

Thus, the OXPHOS chain appears as a promising candidate pathway for CRC progression. The OXPHOS system, whose proteins are encoded by both nuclear and mitochondrial DNA (mtDNA), consists of five major protein complexes named complex I, II, III, IV, and V, localized on the inner mitochondrial membrane. Its main function is the generation of ATP [10]. We therefore selected putatively functional single nucleotide polymorphisms (SNPs) in the genes from the pathway, and investigated their possible role in the progression of CRC and the prognosis of patients in a group of 613 German CRC patients.

## Methods

### Study population

The 613 patients included in the study belonged to the population-based PopGen project in Schleswig-Holstein (Germany) [11]. All individuals were of German origin, i.e. with both parents born in Germany. Patients fulfilling either the clinical Amsterdam or Bethesda criteria for hereditary non-polyposis colorectal cancer (HNPCC) were excluded from the study, as were patients with a history of malignant disease or inflammatory bowel disease. For a full description of the characteristics of the 613 patients see Table 1 and Castro et al. [12]. The work has been approved by the corresponding ethical committees and subjects gave informed consent to the work.

**Table 1:** Characteristics of the 613 CRC patients at the time of diagnosis

**Parameter**	**Number of patients**	**% of patients**
**Gender**		
Male	325	53.0
Female	284	46.3
Unknown	4	0.7
**Age at diagnosis (year)**		
<65	341	55.6
≥65	272	44.4
**Histopathologic grade (G)**		
G1	20	3.3
G2	478	77.9
G3	98	16.0
G4	1	0.2
Gx	16	2.6
**Pretherapeutic UICC TNM stage**		
I	161	26.3
II	156	25.4
III	149	24.3
IV	133	21.7
Unknown	14	2.3
**Primary tumour size (T)**		
T0	6	1.0
T1	76	12.3
T2	138	22.5
T3	327	53.3
T4	58	9.4
Tis or Tx	9	1.5
**Regional lymph node involvement (N**)		
N-	354	57.7
N+	255	41.6
Nx	4	0.7
**Distant metastasis (M)**		
M-	478	78.0
M+	135	22.0
**Localisation**		
Rectum	327	53.3
Colon	286	46.7
**Adjuvant therapy**		
Any^a^	250	40.8
None	282	46.0
Unknown	81	13.2
**Pretreatment**		
Any^b^	68	11.1
None	517	84.3
Unknown	28	4.6

a Comprises radiotherapy (n = 13), chemotherapy (n = 86), radio-chemotherapy (n = 59), immunotherapy (n = 3), chemotherapy plus 5FU/FA (n = 68), radio-chemotherapy plus 5FU/FA (n = 18), and other (n =3).

b Comprises radiotherapy (n = 5) and radio-chemotherapy (n = 63).

### Gene and SNP selection

Candidate genes in the OXPHOS pathway (map00190 in the KEGG database [13]) were selected based on a recently published meta-analysis of GEP studies on CRC prognosis [3]. Three out of the seven genes reported in two GEP studies had SNPs in the putative regulatory regions (ATP5C1 from complex V, COX5B and COX6B1 from complex IV). Two additional genes with SNPs in putative regulatory regions, each reported in one GEP study, were also included, namely NDUFS2 from complex I and UQCRB from complex III. With these selection criteria, the four complexes from the OXPHOS chain which allow flowing of protons between the mitochondrial matrix and the intermembrane space, resulting into energy generation in form of ATP, were included in the study. Finally, SNPs in the genes GAPDH and HSPD1 were also selected, since both genes might define the cellular bioenergetic activity of the mitochondria [6].

SNPs within the set of candidate genes (Table 2 and Additional file 1: Table S1) were selected according to the following criteria: minor allele frequency (MAF) > 0.05 in HapMap CEPH population (Utah residents with ancestry from northern and western Europe - CEU), and location in potentially regulatory gene regions (5′ and 3′-UTR, promoter or non-synonymous coding SNPs). In regions with several SNPs in high linkage disequilibrium (LD), tagSNPs were selected for genotyping. LD between the SNPs was calculated using Haploview [14]. The promoter SNP rs2802460 in ATP5C1 was reported to regulate expression of the gene in lymphoblastoid cell lines (eSNPs) on a genome-wide association study of global gene expression [15]. Predictions of the possible impact of amino acid substitutions on the structure and function of the protein were performed with PolyPhen2 [16].

**Table 2:** SNPs evaluated in the study

**Gene**	**Complex**	**SNP**	**Major/minor allele**	**Location**	**MAF**
NDUFS2	I	rs33941127	C/T	Promoter	T - 0.21
NDUFS2	I	rs3813623	G/T	5′-UTR	T - 0.15
NDUFS2	I	rs11538340	C/A	Coding P20T	A - 0.06
NDUFS2	I	rs11576415	C/G	Coding P352A	G - 0.09
NDUFS2	I	rs1136224	A/G	3′-UTR	G - 0.17
UQCRB	III	rs7836698	C/T	3′-UTR	T - 0.42
UQCRB	III	rs10504961	C/T	3′-UTR	T - 0.50
COX5B	IV	rs11904110	T/C	Promoter	C – 0.06
COX6B1	IV	rs6510502	A/C	Promoter	C - 0.13
COX6B1	IV	rs10420252	G/A	5′-UTR	A - 0.10
ATP5C1	V	rs11255367	G/A	Promoter	A - 0.12
ATP5C1	V	rs2802460	T/C	Promoter	C - 0.26
ATP5C1	V	rs4655	T/C	3′-UTR	C - 0.36
GAPDH		rs7971637	C/T	Promoter	T - 0.19
GAPDH		rs1136666	C/G	5′-UTR	G - 0.26
HSPD1		rs1116734	C/G	Promoter	G - 0.33

Two additional SNPs were genotyped, but showed only one allele in the 613 samples: rs2071038 (COX5B) and rs955 (HSPD1).

MAF, minor allele frequency in the 613 CRC patients investigated; NDUFS2, NADH dehydrogenase (ubiquinone) Fe-S protein 2, 49kDa (NADH-coenzyme Q reductase); UQCRB, ubiquinol-cytochrome c reductase binding protein; COX5B, cytochrome c oxidase subunit Vb; COX6B1, cytochrome c oxidase subunit VIb, polypeptide 1; ATP5C1, ATP synthase, H + transporting, mitochondrial F1 complex, gamma polypeptide 1; GAPDH, glyceraldehyde-3-phosphate dehydrogenase; HSPD1, heat shock 60kDa protein 1 (chaperonin).

### Genotyping

The 613 DNAs used in the study were extracted from peripheral venous blood using the FlexiGene chemistry (Qiagen, Hilden, Germany) according to the manufacturer’s protocols. From them, 272 (44.4%) samples were subjected to genome-wide amplification using the illustra GenomiPhi V2 DNA Amplification Kit (GE Healthcare, Freiburg, Germany) according to manufacturer’s instructions. Genotyping was performed using 5 ng of either genomic or whole-genome amplified DNA in 384-well plate format, using KASPar assays on demand (KBiosciences, Hoddesdon, UK) and following the manufacturer’s protocols. Average genotyping call rate was 98.8%. All polymorphisms were in Hardy-Weinberg equilibrium.

### Statistical analysis

SAS software version 9.2 (SAS Institute) was used in all analysis. Unadjusted association between the genotyped markers and patient characteristics were evaluated by chi-square test. Odds ratios (ORs) with 95% confidence intervals (CIs) were estimated using logistic regression. Effect of the different genotypes on CRC survival was evaluated using Kaplan-Meier method and was compared using log-rank testing. Analysis of different parameters for prognostic significance was done by univariate and multivariate Cox proportional hazard models. P values < 0.05 were considered statistically significant. Follow-up time was calculated from the date of CRC diagnosis to the CRC specific death or death by any cause or to the end of follow-up (date of last contact with the treating physician). Taking into account the possible biological effects of the selected genes/variants on cancer progression and a possible correlation between the variants, correction for multiple comparisons was excluded.

## Results

### SNP selection

A total of 29 SNPs were identified in the regions of interest (5′- and 3′-UTR, promoter and non-synonymous coding SNPs) of the seven genes NDUFS2, UQCRB, COX5B, COX6B1, ATP5C1, GAPDH and HSPD1 ( Additional file 1: Table S1). Out of them, 18 SNPs were selected for the association studies, covering all gene regions with a putative regulatory function.

### Association with survival

**Figure 1:**
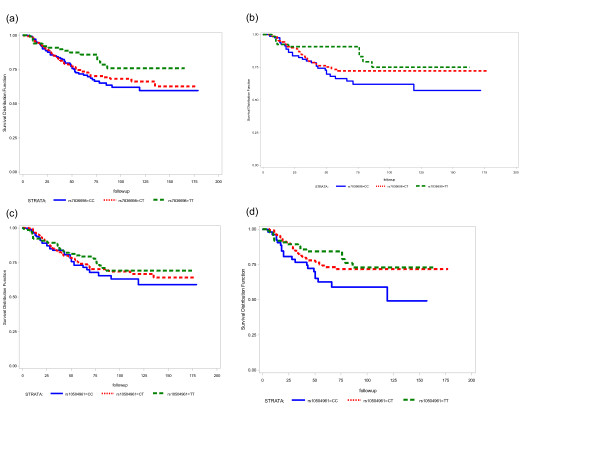
Kaplan-Meier estimates of CRC specific survival (months) according to UQCRB genotypes, (a) rs7836698, all CRC patients (n = 606), log rank p value = 0.07, (b) rs7836698, all patients diagnosed with colon cancer (n = 324), log rank p value = 0.05, (c) rs10504961, all CRC patients (n = 606), log rank p value = 0.55, and (d) rs10504961, all patients diagnosed with colon cancer (n = 324), log rank p value = 0.06.

The two polymorphisms in the 3′-UTR region of UQCRB (complex III) showed association with CRC survival (Table 3 and Additional file 1: Table S2). The marker rs7836698 was statistically significantly associated with a decreased risk of death due to CRC (HR = 0.53, 95% CI 0.31–0.91 for TT), or due to any cause (HR = 0.52, 95% CI 0.32–0.85). For rs10504961, only the association with overall survival was statistically significant (HR = 0.64, 95% CI 0.42–0.99 for TT). Interestingly, for both UQCRB polymorphisms, the TT carriers were more likely to develop tumours in the colon than in the rectum, with ORs of 2.02 (95% CI 1.24–3.28) for rs7836698 (TT vs. CC) and 1.74 (95% CI 1.08–2.78) for rs10504961 (TT vs. CC). When survival was analysed according to the cancer site, both SNPs showed association exclusively in the group of patients with cancer located in the colon (HR for overall survival 0.42, 95% CI 0.22–0.82 for rs7836698 TT carriers and 0.46, 95% CI 0.25–0.83 for rs10504961 TT carriers), but not in the rectum (Table 3). The Kaplan-Meier survival curves representing the survival rates of the patients according to their genotypes, as well as the P values for the log-rank test are presented in Figure 1.

**Table 3:** Association of two polymorphisms in UQCRB (complex III) with survival

**SNP**	**Genotype**		**Cause of death: CRC**			**Cause of death: any**		
		**No.**	**Deaths (%)**	**HR** **(95% CI)**	**P value**	**Deaths (%)**	**HR** **(95% CI)**	**P value**
**rs7836698** **All patients**	CC	203	54 (26.6)	1.00		69 (34.0)	1.00	
	CT	297	77 (25.9)	0.90 (0.63–1.28)	0.55	95 (32.0)	0.87 (0.64–1.20)	0.41
	TT	106	18 (17.0)	**0.53** **(0.31–0.91)**	**0.02**	23 (21.7)	**0.52** **(0.32–0.85)**	**0.01**
**rs7836698** **Colon**	CC	97	30 (30.9)	1.00		37 (38.1)	1.00	
	CT	159	38 (23.9)	0.74 (0.45–1.21)	0.23	47 (29.6)	0.75 (0.48–1.18)	0.21
	TT	69	10 (14.5)	**0.42** **(0.21–0.87)**	**0.02**	13 (18.8)	**0.42** **(0.22–0.82)**	**0.01**
**rs7836698** **Rectum**	CC	105	23 (21.9)	1.00		31 (29.5)	1.00	
	CT	137	39 (28.5)	1.13 (0.67–1.90)	0.65	48 (35.0)	1.03 (0.65–1.63)	0.89
	TT	37	8 (21.6)	0.73 (0.32–1.71)	0.47	10 (27.0)	0.70 (0.33–1.47)	0.34
**rs10504961** **All patients**	CC	134	34 (25.4)	1.00		47 (35.1)	1.00	
	CT	319	78 (24.4)	0.88 (0.58–1.33)	0.54	97 (30.4)	0.79 (0.55–1.13)	0.19
	TT	150	36 (24.0)	0.76 (0.47–1.24)	0.28	42 (28.0)	**0.64** **(0.42–0.99)**	**0.05**
**rs10504961** **Colon**	CC	64	22 (34.4)	1.00		27 (42.2)	1.00	
	CT	168	38 (22.6)	0.61 (0.35–1.05)	0.07	48 (28.6)	0.62 (0.38–1.01)	0.06
	TT	92	18 (19.6)	**0.47** **(0.25–0.90)**	**0.02**	22 (23.9)	**0.46** **(0.25–0.83)**	**0.01**
**rs10504961** **Rectum**	CC	70	12 (17.1)	1.00		20 (28.6)	1.00	
	CT	149	39 (26.2)	1.33 (0.69–2.54)	0.39	48 (32.2)	0.98 (0.58–1.66)	0.95
	TT	58	18 (31.0)	1.41 (0.66–2.97)	0.37	20 (34.5)	0.96 (0.51–1.81)	0.89

HR, hazard ratio; CI, confidence interval.

We examined also whether inclusion of other variables associated with CRC survival affected the parameter estimates for the UQCRB polymorphisms. The risk of death for carriers of the TT genotype at rs7836698 remained statistically significantly decreased after adjustment for age at diagnosis and TNM stage. Among patients with tumour located in the colon, the hazard ratios were similar both in the uni- and multivariate analyses for rs7836698 and rs10504961, although the statistical significance was sometimes lost.

### Association with CRC progression

Three of the 16 SNPs investigated showed an association with one of the different components that form the UICC stage (T, N and M) in a dominant model. Two SNPs in COX6B1 (complex IV) showed an association with the presence of affected lymph nodes (rs6510502, OR = 1.75, 95% CI 1.20–2.57; rs10420252, OR = 1.68, 95% CI 1.11–2.53). The polymorphism rs6510502 was also associated with distant metastasis (OR = 1.67, 95% CI 1.09–2.56). The marker rs7971637 in GAPDH was associated with tumour stage T3/T4 (OR = 1.60, 95% CI 1.11–2.30).

## Discussion

In a homogeneous Northern German population of 613 CRC patients, we observed several associations of polymorphisms in the OXPHOS chain with survival and progression of the disease. Mainly, two SNPs in the 3′-UTR region of UQCRB (complex III), rs7836698 and rs10504961, were associated with survival. The two polymorphisms were in relatively strong linkage disequilibrium (D′ = 0.98, r^2^ = 0.65 in the 613 samples), explaining the association observed with the two SNPs. Added to that, the two studied polymorphisms in COX6B1 (complex IV), rs6510502 in the promoter and rs10420252 in the 5′-UTR, were associated with lymph node metastasis, and rs6510502 also with distant metastasis. The two markers were in high LD (D′ = 0.97, r^2^ = 0.71 in the 613 samples).

Patients carrying the TT genotype for any of the two SNPs in the UQCRB gene had an increased CRC survival. Interestingly, the association appeared to be restricted to the group of patients with tumour located in the colon. Multivariate analyses confirmed that the association was independent of other variables associated with CRC survival, indicating that both markers might act as independent prognostic markers. In the group of patients with tumour located in the colon the hazard ratios remained low, even though the significance sometimes disappeared when other variables were included. Our results may partially explain the previously reported higher familial risk for colon than for rectal cancer, which was shown to decrease gradually from proximal colon to distal colon and to rectum [17]. Also for survival, differences have been observed between colon and rectal cancer, with rectal cancer having somewhat better survival compared to colon cancer [18].

UQCRB is one of the ten nucleus-encoded subunits of the complex III in the mitochondrial respiratory chain, the *ubiquinol-cytochrome c oxidoreductase*. In a genome-wide analysis of pancreatic cancer with CGH (comparative genomic hybridization) arrays, a DNA copy number variation was identified at the UQCRB locus, and this gene was suggested as a potential diagnostic marker or a therapeutic target [19]. Recently, it has been demonstrated that UQCRB plays a crucial role in the oxygen sensing mechanism that regulates responses to hypoxia. Using the small molecule terpestacin to inhibit UQCRB, tumour angiogenesis was blocked in vivo [20]. COX6B1 is one of the ten nucleus-encoded subunits of the complex IV in the mitochondrial respiratory chain, the cytochrome c oxidase. Mutations in this gene have been linked to severe infantile encephalomyopathy, a phenotype associated with cytochrome c oxidase deficiency [21]. Our results suggest that genetic variation in the regulatory regions of genes in the OXPHOS chain may associate with CRC progression. Whether such variation affects the gene expression pattern, impairs the function of mitochondria and leads to cancer development and progression remains to be elucidated.

One of the polymorphisms we investigated, rs2802460, located on the promoter of ATP5C1 (complex V), was reported to regulate expression of the gene in lymphoblastoid cell lines in a genome-wide association study of global gene expression [15]. However, this SNP did not show any association with CRC survival or disease progression. A recent report has evaluated the role of 376 tagSNPs (one included in our study, rs11576415 in NDUFS2) in 78 nuclear-encoded mitochondrial genes (including ATP5C1, NDUFS2, and UQCR) in prostate cancer risk, without clear evidence of association [22]. Another recent large association study included variants from 90 genes involved in oxidative phosphorylation (including ATP5C1, COX5B, COX6B1, GAPDH, and NDUFS2) and tested their association with epithelial ovarian cancer risk; no indication of association was found [23]. To our knowledge, this is the first association study of variants in the respiratory chain with CRC survival and progression.

## Conclusions

Our findings suggest a possible influence of genes in the OXPHOS chain in CRC survival and progression, and support the newly discovered Warburg’s hypothesis. This is the first report of a possible role of genes in the respiratory chain in the prognosis of CRC patients, but further independent studies are clearly needed to elucidate the validity of the results.

## Competing interests

The authors declare that they have no competing interests.

## Author’s contributions

JL, KH, and AF conceived and designed the study. JL carried out the molecular genetic studies. JL wrote the initial manuscript. MB performed the statistical analyses. KH and AF provided oversight and conceptual guidance to the project. MB, WS, HK, SB, JH, KH, AF and CS contributed to the final manuscript. WS, HK, HA, JB, JE, SB, TB, SS, JH, and CS participated in the clinical survey and sample collection. All authors read and approved the final manuscript.

## Pre-publication history

The pre-publication history for this paper can be accessed here:

http://www.biomedcentral.com/1471-2350/13/31/prepub

## Supplementary Material

Additional file 1 **Table S1. **SNPs in the regions of interest (5′- and 3′-UTR, promoter region and non-synonymous coding SNPs) with MAF > 0 in European population. **Table S2.** Association of the 16 investigated polymorphisms with survival. Click here for file
