# Supplementary material for: Polymorphisms in the mitochondrial oxidative phosphorylation chain genes as prognostic markers for colorectal cancer
Source: BMC Med Genet. 2012 Apr 30;13:31. doi: 10.1186/1471-2350-13-31 (PMC3420261; doi:10.1186/1471-2350-13-31)
Supplement: Additional file 1 — Table S1. SNPs in the regions of interest (5′- and 3′-UTR, promoter region and non-synonymous coding SNPs) with MAF > 0 in European population. Table S2. Association of the 16 investigated polymorphisms with survival. [file 1471-2350-13-31-S1.pdf]

Supplementary Table 1. SNPs in the regions of interest (5'- and 3'-UTR, promoter region and non-synonymous coding SNPs) with MAF > 0 in European population.

**Gene**	**SNP**	**Location**	**MAF**	**HapMap CEU**	**tagSNP**	**Functional effect#**
NDUFS2	rs33941127*	Promoter	0,12			
NDUFS2	rs34378188	Promoter	0,33			
NDUFS2	rs34448954	Promoter	0,06			
NDUFS2	rs3813623*	5´-UTR	0,09	X	X	
NDUFS2	rs11538340*	P20T	0,13			benign
NDUFS2	rs11576415*	P352A	0,10	X	X	probably damaging
NDUFS2	rs1136224*	3´-UTR	0,20	X	X	
UQCRB	rs7836698*	3´-UTR	0,35			
UQCRB	rs7002575	3´-UTR	0,45	X		
UQCRB	rs10504961*	3´-UTR	0,45	X	X	
UQCRB	rs2292836	3´-UTR	0,46	X		
COX5B	rs2071038*	Promoter	0,008	X	X	
COX5B	rs17022045	Promoter	0,11			
COX5B	rs11904110*	Promoter	0,11	X	X	
COX6B1	rs6510502*	Promoter	0,11	X	X	
COX6B1	rs10420252*	5´-UTR	0,08	X	X	
ATP5C1	rs11255367*	Promoter	0,09	X	X	
ATP5C1	rs2802460*	Promoter	0,27	X	X	eSNP
ATP5C1	rs17553878	Promoter	0,41			
ATP5C1	rs2070594	Promoter	0,27	X		
ATP5C1	rs4655*	3´-UTR	0,34	X	X	
GAPDH	rs6489721	Promoter	0,27	X		
GAPDH	rs7971637*	Promoter	0,25	X	X	
GAPDH	rs1136666*	5´-UTR	0,37	X	X	
GAPDH	rs1803622	3´-UTR	0,43			
HSPD1	rs2305560	Promoter	0,17	X		
HSPD1	rs16866259	Promoter	0,04			
HSPD1	rs1116734*	Promoter	0,28			
HSPD1	rs955*	3´-UTR	0,12			

* 18 SNPs selected for the association study. # *eSNP*: SNP reported to regulate expression of the gene in lymphoblastoid cell lines (eSNPs) in a genome-wide association study of global

gene expression [1]; *benign, possibly damaging, probably damaging, unknown*: prediction of the possible impact of amino acid substitutions on the structure and function of the protein according to PolyPhen2 [2].

References:

1. Dixon AL, Liang L, Moffatt MF, Chen W, Heath S, Wong KC, Taylor J, Burnett E, Gut I, Farrall M *et al*: **A genome-wide association study of global gene expression.** *Nat Genet* 2007, **39**(10):1202-1207.
2. Adzhubei IA, Schmidt S, Peshkin L, Ramensky VE, Gerasimova A, Bork P, Kondrashov AS, Sunyaev SR: **A method and server for predicting damaging missense mutations.** *Nat Methods* 2010, **7**(4):248-249.

Supplementary Table 2. Association of the 16 investigated polymorphisms with survival.

SNP	Genotype		Cause of death: CRC			Cause of death: any		
		**No.**	**Deaths (%)**	**HR (95% CI)**	**P value**	**Deaths (%)**	**HR (95% CI)**	**P value**
**rs33941127**	CC	379	90 (23.75)	1.00		108 (28.50)	1.00	
**NDUFS2**	CT	201	52 (25.87)	1.17 (0.83-1.66)	0.37	71 (35.32)	1.30 (0.95-1.77)	0.10
	TT	28	5 (17.86)	0.83 (0.34-2.05)	0.69	7 (25.00)	0.97 (0.45-2.09)	0.94
**rs3813623**	GG	438	108 (24.66)	1.00		138 (31.51)	1.00	
**NDUFS2**	GT	155	38 (24.52)	0.98 (0.67-1.42)	0.90	45 (29.03)	0.92 (0.65-1.29)	0.62
	TT	17	2 (11.76)	0.46 (0.11-1.88)	0.28	4 (23.53)	0.74 (0.27-1.99)	0.55
**rs11538340**	CC	534	135 (25.28)	1.00		166 (31.09)	1.00	
**NDUFS2**	AC	74	11 (14.86)	0.53 (0.27-1.04)	0.07	19 (25.68)	0.83 (0.50-1.37)	0.46
	AA	2	1 (50.00)	3.10 (0.43-22.3)	0.26	1 (50.00)	2.68 (0.37-19.21)	0.33
**rs11576415**	CC	500	118 (23.60)	1.00		153 (30.60)	1.00	
**NDUFS2**	CG	106	29 (27.36)	1.19 (0.79-1.81)	0.41	33 (31.13)	1.05 (0.71-1.53)	0.82
	GG	4	2 (50.00)	2.51 (0.62-10.2)	0.20	2 (50.00)	1.86 (0.46-7.53)	0.38
**rs1136224**	AA	422	104 (24.64)	1.00		126 (29.86)	1.00	
**NDUFS2**	AG	160	39 (24.38)	1.04 (0.72-1.51)	0.84	54 (33.75)	1.21 (0.87-1.67)	0.25
	GG	23	5 (21.74)	0.96 (0.39-2.36)	0.93	7 (30.43)	1.13 (0.53-2.42)	0.76
**rs7836698**	CC	203	54 (26.6)	1.00		69 (34.0)	1.00	
**UQCRB**	CT	297	77 (25.9)	0.90 (0.63-1.28)	0.55	95 (32.0)	0.87 (0.64-1.20)	0.41
	TT	106	18 (17.0)	**0.53 (0.31-0.91)**	**0.02**	23 (21.7)	**0.52 (0.32-0.85)**	**0.01**
**rs10504961**	CC	134	34 (25.4)	1.00		47 (35.1)	1.00	
**UQCRB**	CT	319	78 (24.4)	0.88 (0.58-1.33)	0.54	97 (30.4)	0.79 (0.55-1.13)	0.19
	TT	150	36 (24.0)	0.76 (0.47-1.24)	0.28	42 (28.0)	**0.64 (0.42-0.99)**	**0.05**
**rs11904110**	TT	545	135 (24.77)	1.00		168 (30.83)	1.00	
**COX5B**	CT	62	12 (19.35)	0.85 (0.46-1.57)	0.59	18 (29.03)	1.01 (0.61-1.69)	0.97
	CC	4	0 (0.00)	-	-	0 (0.00)	-	-
**rs6510502**	AA	469	113 (24.09)	1.00		144 (30.70)	1.00	
**COX6B1**	AC	120	30 (25.00)	1.11 (0.74-1.68)	0.61	36 (30.00)	1.05 (0.72-1.53)	0.80
	CC	19	6 (31.58)	1.16 (0.51-2.65)	0.72	7 (36.84)	1.09 (0.51-2.33)	0.82

**rs10420252**	GG	496	121 (24.40)	1.00		151 (30.44)	1.00	
**COX6B1**	AG	102	24 (23.53)	1.00 (0.64-1.55)	0.99	31 (30.39)	1.02 (0.69-1.51)	0.92
	AA	11	3 (27.27)	1.14 (0.36-3.59)	0.82	5 (45.45)	1.58 (0.65-3.89)	0.32
**rs11255367**	GG	466	108 (23.18)	1.00		137 (29.40)	1.00	
**ATP5C1**	AG	128	37 (28.91)	1.27 (0.87-1.85)	0.22	46 (35.94)	1.18 (0.84-1.66)	0.35
	AA	8	3 (37.50)	1.81 (0.57-5.70)	0.31	3 (37.50)	1.47 (0.47-4.63)	0.51
**rs2802460**	TT	329	76 (23.10)	1.00		98 (29.79)	1.00	
**ATP5C1**	CT	241	61 (25.31)	1.11 (0.79-1.57)	0.54	75 (31.12)	1.08 (0.79-1.47)	0.63
	CC	37	11 (29.73)	1.45 (0.75-2.82)	0.27	12 (32.43)	1.15 (0.60-2.21)	0.68
**rs4655**	TT	251	56 (22.31)	1.00		73 (29.08)	1.00	
**ATP5C1**	CT	275	73 (26.55)	1.21 (0.85-1.73)	0.29	93 (33.82)	1.23 (0.90-1.68)	0.20
	CC	78	19 (24.36)	1.13 (0.66-1.92)	0.66	21 (26.92)	0.94 (0.56-1.55)	0.79
**rs7971637**	CC	397	89 (22.42)	1.00		113 (28.46)	1.00	
**GAPDH**	CT	189	54 (28.57)	1.39 (0.99-1.96)	0.06	68 (35.98)	1.35 (0.99-1.83)	0.06
	TT	21	5 (23.81)	1.24 (0.50-3.05)	0.64	6 (28.57)	0.94 (0.39-2.32)	0.90
**rs1136666**	CC	338	79 (23.37)	1.00		100 (29.59)	1.00	
**GAPDH**	CG	224	57 (25.45)	1.18 (0.83-1.66)	0.36	73 (32.59)	1.15 (0.84-1.57)	0.38
	GG	45	12 (26.67)	1.24 (0.66-2.34)	0.51	14 (31.11)	1.12 (0.63-2.01)	0.70
**rs1116734**	CC	266	65 (24.44)	1.00		86 (32.33)	1.00	
**HSPD1**	CG	278	64 (23.02)	0.91 (0.64-1.30)	0.60	82 (29.50)	0.89 (0.65-1.21)	0.46
	GG	59	18 (30.51)	1.12 (0.65-1.91)	0.69	18 (30.51)	0.85 (0.51-1.44)	0.55

HR, hazard ratio; CI, confidence interval
